# Exploring Awareness Levels of Diabetic Ketoacidosis Risk Among Patients with Diabetes: A Cross-Sectional Study

**DOI:** 10.3390/clinpract14060211

**Published:** 2024-12-12

**Authors:** Elhassan Hussein Eltom, Abdulrahman Omar A. Alali, Rakan Khalid Marzouq Alanazi, Ali Ahmad M. Alanazi, Meshal Ahmed Abdullah Albalawi, Saud Alraydh N. Alanazi, Mansour Sarhan G. Alanazi, Abdelnaser A. Badawy, Naglaa Mokhtar, Manal S. Fawzy

**Affiliations:** 1 Department of Pharmacology, Faculty of Medicine, Northern Border University, Arar 91431, Saudi Arabia; alhassan.abdallah@nbu.edu.sa; 2 Faculty of Medicine, Northern Border University, Arar 91431, Saudi Arabia; st202101039@stu.nbu.edu.sa (A.O.A.A.); st202101599@stu.nbu.edu.sa (R.K.M.A.); st202000133@stu.nbu.edu.sa (A.A.M.A.); st202100735@stu.nbu.edu.sa (M.A.A.A.); st202000220@stu.nbu.edu.sa (S.A.N.A.); 3 Endocrinology Department, North Medical Tower of Northern Border Region, Arar 73241, Saudi Arabia; malanazi398@moh.gov.sa; 4 Internal Medicine, Northern Borders Health Cluster, Arar 91431, Saudi Arabia; 5 Department of Medical Biochemistry, Faculty of Medicine, Northern Border University, Arar 91431, Saudi Arabia; abdelnaser.ali@nbu.edu.sa (A.A.B.); naglaa.ibrahim@nbu.edu.sa (N.M.); 6 Medical Biochemistry and Molecular Biology Department, Faculty of Medicine, Mansoura University, Mansoura 35516, Egypt; 7 Center for Health Research, Northern Border University, Arar 91431, Saudi Arabia

**Keywords:** DKA, diabetes type 1, diabetes type 2, awareness, Northern Borders region

## Abstract

**Background/Objectives**: Diabetic ketoacidosis (DKA) is a critical complication of diabetes mellitus, posing significant health. While global studies have indicated a concerning lack of awareness regarding DKA among patients with diabetes, research specific to the northern area of Saudi Arabia remains limited. This study aims to explore the level of knowledge and awareness of DKA among patients with diabetes residing in the local region. **Methods**: A cross-sectional analysis was conducted utilizing a non-probability convenient sampling technique, with 339 participants recruited from March to August 2024. Data were gathered through a self-administered pre-validated questionnaire distributed via different social media platforms to assess demographic characteristics and awareness levels relating to DKA, including knowledge of its symptoms, causes, and treatment options. **Results**: Although there was moderate awareness of DKA, with 68.4% having heard of the condition, two-thirds of the participants exhibited significant gaps in overall knowledge. Among those aware, 76.3% recognized DKA as an emergency requiring immediate medical intervention. At the same time, 64.6% understood the causes of DKA, and only 25.6% identified insulin deficiency as a major contributing factor. Although 62.5% felt knowledgeable about treatment, 66.0% incorrectly identified oral sugar as a DKA treatment. Notably, 30.1% cited social media as their main information source. Age emerged as an essential factor impacting knowledge, with younger participants (ages 18–30) demonstrating higher awareness than older individuals. Additionally, single participants displayed a higher percentage of good knowledge than married participants (*p* = 0.000). Non-working individuals showed better overall knowledge about DKA (*p* = 0.002). The duration of diabetes did not show a significant association with knowledge levels about DKA across the various duration categories. **Conclusions**: The present findings underscore a substantial knowledge gap concerning DKA among the local community, highlighting a critical need for targeted public health educational interventions.

## 1. Introduction

Diabetes mellitus (DM) encompasses a group of metabolic disorders characterized by elevated blood glucose levels. This condition can result from insulin resistance, reduced insulin effectiveness, or increased levels of counter-regulatory hormones [1]. One of the most critical and potentially life-threatening complications of diabetes is diabetic ketoacidosis (DKA) [2]. It typically manifests when insufficient insulin is produced in the body, preventing glucose from being utilized as an energy source. This deficiency leads to increased blood glucose levels, elevated circulating fatty acids, and the production of ketones, culminating in DKA [3]. The primary factors contributing to DKA development include insufficient insulin and missed insulin doses. Three hallmark features characterize DKA: anion gap metabolic acidosis, ketosis, and hyperglycemia. Biochemically, DKA is defined as an increase in serum ketone concentration greater than 5 mEq/L, blood glucose levels exceeding 250 mg/dL, and arterial pH below 7.3 [3,4].

The most common early symptoms of DKA include an insidious increase in polydipsia (excessive thirst) and polyuria (frequent urination). Other signs and symptoms may include malaise, generalized weakness, nausea, vomiting, abdominal pain, decreased appetite, rapid weight loss, and altered consciousness [5]. Early recognition and prompt management of DKA are essential for preventing severe outcomes, including coma and death [6].

Notably, between 25 and 40% of individuals with type 1 diabetes mellitus (T1DM) may present with DKA at initial diagnosis, and it can also affect at least 34% of people with type 2 diabetes [7]. The most common scenarios for DKA occurrence include underlying or concomitant infection (40%), missed or disrupted insulin treatments (25%), and newly diagnosed, previously unknown diabetes (15%) [8].

Globally, several studies have documented concern about the lack of awareness regarding DKA among patients with diabetes [9,10,11]. For instance, a study conducted by Hepprich and colleagues found that 32% of patients with T1DM were not familiar with the term ‘diabetic ketoacidosis’, and 45% were unaware of its causes [11]. A study in Sudan reported that just 39.1% and 26.4% of study participants recognized the loss of consciousness and death as complications of this condition, respectively [12]. At the same time, a similar study in Turkey reported that just 28.6% of patients recognized the severity of this condition [13]. These findings point to a significant gap in knowledge, which may lead to delayed diagnosis and increased morbidity and mortality.

In the Saudi context, limited research has focused explicitly on DKA awareness. However, existing studies examining diabetes knowledge and self-management practices among Saudi patients indicate a suboptimal awareness of diabetes complications [14,15]. A study conducted in Riyadh, Saudi Arabia, found that 64.7% of caregivers of diabetic children reported knowing about DKA, while 35.3% did not [16]. Another study found that 38.7% of participants have poor awareness regarding DKA complications, and 67.3% have poor knowledge concerning its management [14].

These findings suggest a need for improved education and awareness programs. Given the potential severity of DKA and the apparent knowledge gaps, there is a clear need for targeted public health interventions to improve awareness and understanding of this condition among patients with diabetes and their caregivers. Therefore, this study aims to assess the awareness level of DKA among patients with diabetes in the general population of the authors’ region, with the goal of informing future educational initiatives and improving patient outcomes.

## 2. Materials and Methods

### 2.1. Study Design

This cross-sectional study was conducted from March to August 2024 to explore the level of awareness of DKA among patients with diabetes residing in the northern region of Saudi Arabia near the borders with Iraq.

### 2.2. Participants

A non-probability, cluster-convenient sampling technique was employed for participant recruitment. Male and female patients diagnosed with either T1DM or T2DM from the general population residing in the specified region and willing to participate were included. Exclusion criteria encompassed healthcare providers and individuals living outside the designated area.

### 2.3. Ethical Considerations

The research proposal was approved by the local bioethical committee at Northern Border University (approval reference: 8/24/H, dated 31 January 2024). Informed digital consent was obtained from all participants prior to their participation. Participant information was anonymized to ensure privacy, with no personal identifiers collected. Access to the gathered data was restricted solely to the research team members to maintain confidentiality.

### 2.4. Sample Size Calculation

The sample size was computed using a formula for determining the sample size for a cross-sectional study, ‘[*n* = Z^2^p (1 − p)/d^2^]’, where (*n*) represents the sample size, (p) is the estimated proportion (32.6%), (Z) corresponds to 1.96 for a 95% confidence level, and (d) is the acceptable margin of error (0.05) [17]. Based on this calculation, the minimum required sample size was determined to be 339 participants.

### 2.5. Data Collection

Data were collected using a self-administered, pre-validated questionnaire distributed via various social media platforms [14]. Two experts translated the questionnaire into Arabic and validated it for cultural appropriateness prior to distribution. Ten individuals were also included in a pilot study to assess the questionnaire’s clarity and comprehensibility; this pilot sample was excluded from the final analysis. The authors continued data collection until they successfully gathered responses from 339 participants, reflecting the calculated sample size. As such, the participation rate is regarded as 100% for those who completed the survey among the study responders. The questionnaire included demographic questions regarding residency, age, sex, nationality, marital status, and occupation, along with items assessing knowledge and awareness of DKA, including treatment approaches, symptom recognition, and sources of information related to diabetes and DKA.

### 2.6. Statistical Analysis

Statistical analysis was performed using the Statistical Package for Social Sciences (SPSS) version 24.0 (IBM SPSS Statistics, Armonk, NY, USA). Data distribution was assessed to determine the appropriate statistical tests. Descriptive data were reported as frequencies with percentages and/or mean ± standard deviation (SD).

A scoring system was implemented to evaluate knowledge of DKA, wherein participants received one point for each correct answer. Accumulated points were used to classify knowledge levels as follows: a score of 75% or higher of the total possible points was classified as excellent knowledge, scores between 50% and 74% indicated good knowledge, and scores below 50% indicated inadequate knowledge. For statistical simplicity, ‘good’ and ‘excellent’ knowledge categories were combined into a single ‘good knowledge’ category. Comparisons between knowledge categories (good vs. poor knowledge) were conducted using Chi-square and/or Fisher’s exact tests as appropriate. The significance level was set at a *p*-value of 0.05.

## 3. Results

### 3.1. Study Participants’ Characteristics

A total of 339 participants were included in the study, and their characteristics are summarized in Table 1. Most participants (78.8%) resided in Arar (the capital of the Northern Borders area) and were primarily between 18 and 30 years old (42.2%), with 54.0% of them being males. Most participants (94.4%) were Saudi nationals, and a substantial proportion held university degrees (69.0%). Regarding occupation, many participants (43.1%) reported being unemployed, while 39.5% were employed in governmental institutions.

### 3.2. Knowledge About Diabetic Ketoacidosis

The level of awareness regarding DKA among participants was assessed through a direct question about prior knowledge of the condition. It is noteworthy that approximately 66.7% of participants were unaware of their diabetes type, with only 13.8% identified as T1DM and 19.5% as T2DM.

As presented in Table 2, most participants (68.4%) reported hearing of DKA. Among those aware of DKA, participants were asked to describe the condition. A substantial percentage of participants (76.3%) accurately identified DKA as an emergency diabetes complication requiring urgent treatment. A smaller group (9.9%) perceived DKA as a chronic complication of diabetes that does not necessitate a doctor’s visit and can be managed at home, and 13.8% described DKA as merely one of the symptoms of diabetes that should be accommodated (Table 2).

Participants’ knowledge of the causes/contributing factors to DKA was evaluated, as summarized in Table 3. About two-thirds of participants (64.6%) reported knowing what causes DKA. Among those who acknowledged having knowledge of DKA causes, participants were asked to identify factors that could contribute to its development. The most commonly identified factor, noted by 25.6% of respondents, was the lack of insulin in the blood, primarily due to forgetting to take insulin injections. Meanwhile, a smaller proportion (13.2%) recognized irregular eating and exercise as contributing factors to high blood sugar, which can lead to DKA.

The awareness of symptoms associated with DKA was assessed among the participants, as summarized in Table 4. Of the 339 participants, 61.1% reported familiarity with the symptoms of DKA. Fatigue was the most recognized symptom identified by 87.0% of respondents who realized they were familiar with the DKA symptoms, followed by nausea and blurred vision identified by two-thirds of respondents.

Table 5 illustrates the participants’ understanding of DKA treatment. Of the 339 participants surveyed, 37.5% reported that they did not know how DKA is treated. Among those familiar with DKA treatment, the majority (66.0%) incorrectly identified giving the patient oral sugar and waiting for improvement as an appropriate treatment for DKA. In contrast, 71.2% of participants correctly identified that calling an ambulance and taking the patient to the hospital immediately is an essential step in treating DKA.

### 3.3. Sources of Information Regarding Diabetic Ketoacidosis

The participants’ sources of information regarding diabetes and DKA were assessed, as depicted in Table 6. The findings reveal a diverse range of information sources among the participants, as only one-quarter of respondents reported that healthcare professionals were their primary source of information about diabetes/DKA. A noteworthy one-third of participants identified social media as their primary source of information, and nearly the same proportion indicated that family/friends are the primary sources of information.

### 3.4. Duration of Reported Diabetes Diagnosis Among Study Participants

As depicted in Table 7, about one-third of the participants reported 5 years or less duration, while 50% of participants had diabetes for more than 10 years, with an overall mean duration of reported diabetes as 11.4 ± 7.8 years.

### 3.5. Overall Participants’ Knowledge Regarding Diabetic Ketoacidosis

**Figure 1:**
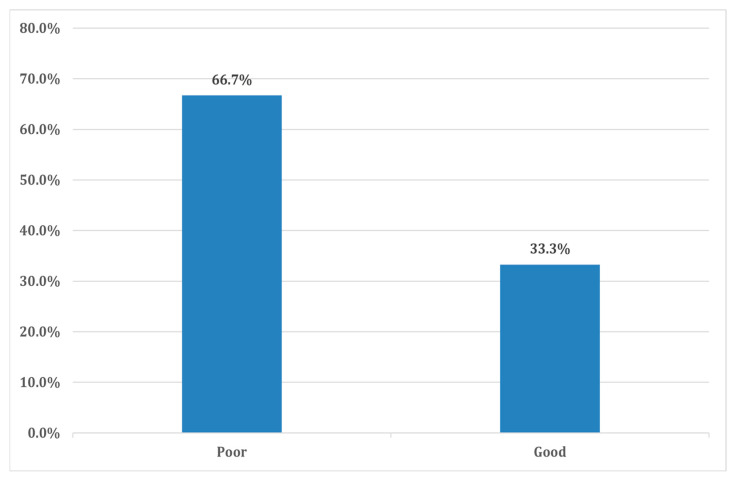
The overall knowledge level among study participants (*n* = 339).

Assessment of the overall knowledge of the study participants revealed a significant knowledge gap regarding DKA among participants, with two-thirds (66.7%, *n* = 226) of individuals exhibiting poor awareness of the condition (Figure 1).

### 3.6. Relationship of Demographic Factors with Overall DKA Knowledge Level

The relationship between demographic factors and the overall knowledge level about DKA among participants was assessed (Table 8). While residency, gender, and nationality did not substantially impact knowledge, age emerged as an essential factor, with younger participants (ages 18–30) demonstrating notably higher awareness than older individuals. Additionally, marital status was linked to knowledge levels, as single participants displayed a higher percentage of good knowledge than married participants (*p* = 0.000). Surprisingly, non-working individuals showed better overall knowledge about DKA (*p* = 0.002).

The assessment of the association between diabetes duration and overall knowledge levels regarding DKA reveals significant findings, particularly regarding previous experience with DKA (Table 9). Participants without previous experience exhibited a significantly higher percentage of poor knowledge (70.6%) than those with prior experience (57.7%) (*p* = 0.020). However, the duration of diabetes did not show a significant association with knowledge levels about DKA across the various duration categories.

## 4. Discussion

The primary objective of this study was to assess the knowledge and awareness of DKA among patients with diabetes in the Northern Borders region of Saudi Arabia while examining demographic variables that impact this understanding. Our findings highlight a significant gap in DKA awareness, underscoring the urgent need for improved public health education on this topic.

The demographic analysis of our study participants indicated that most were residents of Arar City (the capital of the Northern Borders region of Saudi Arabia), aged between 18 and 30 years, and had high educational levels. The gender distribution was relatively balanced, with a slight male predominance, while 43% reported unemployment. A study from the Riyadh region reported a similar demographic distribution regarding age and education, although two-thirds of their participants were male [14]. Furthermore, similar patterns were observed in recent studies conducted in Makkah by Alsaedi et al. [18] and other regions in Saudi Arabia [15], reflecting general trends within the Saudi population.

We acknowledge the importance of distinguishing between T1DM and T2DM, particularly given the differences in age, clinical presentation, and management approaches between these two groups. However, the study findings indicate that approximately 66.7% of participants were unaware of their diabetes type, primarily because they were recruited from the general population through various social media platforms. Only 13.8% of participants identified as T1DM and 19.5% as T2DM. This lack of classification underscores a significant challenge in diabetes management, particularly since a clear understanding of diabetes type is crucial for effective treatment and patient education [19,20,21]. Research indicates that patients’ knowledge of their diabetes type is associated with better self-management practices and improved health outcomes [21]. Moreover, individuals with T1DM often experience specific complications such as DKA due to their absolute insulin deficiency, while those with T2DM typically manage their condition with lifestyle modifications and medications aimed at improving insulin sensitivity [22]. The high percentage of participants who did not know their diabetes type highlights an urgent need for enhanced public education about diabetes. Efforts to improve awareness and knowledge can help guide patients in recognizing the symptoms and risk factors associated with their condition, potentially decreasing the incidence of acute complications [15]. This study’s findings reflect the necessity for tailored educational interventions that effectively communicate the differences between T1DM and T2DM.

Awareness of DKA among our participants was moderate; approximately two-thirds reported prior knowledge of the condition. This contrasts with a Makkah-based study where only one-third demonstrated adequate awareness [17]. Among those aware, nearly three-quarters recognized DKA as an emergency complication of diabetes, while 42.5% identified it correctly as a medical emergency in the northern and western region study [23]. Conversely, only 9.9% believed that DKA could be managed at home, and 13.8% erroneously considered DKA as merely a symptom of diabetes.

Our research further revealed that two-thirds of participants knew the causes of DKA, with one-quarter attributing it primarily to insulin deficiency. The remaining participants suggested irregular lifestyle choices as contributing factors. These results align with findings from the Makkah study, where one-third of their participants correctly identified the missing insulin injections as a primary cause [18], while another one-third recognized it in the northern and western region study by Hassan et al. [15]. Notably, a study from Switzerland reported a higher awareness level, with about two-thirds of respondents correctly identifying missed insulin injections as a common cause of DKA [11].

Regarding DKA symptoms, about two-thirds of respondents reported familiarity, with fatigue being the most recognized symptom, followed by nausea and blurred vision. In contrast, the Swiss study revealed that approximately half of the participants could not identify any symptoms of DKA [11].

Despite a moderate level of DKA awareness, concerns remain regarding knowledge of treatment. Around 62% of participants indicated awareness of DKA treatment, but 66% mistakenly believed it involved administering oral sugar. This contrasts sharply with the Riyadh study, where only 6% had accurate knowledge about DKA treatment [14].

Regarding information sources, our findings indicate that social media and family/friends were the primary channels for information, while one-quarter of the participants relied on healthcare professionals for knowledge about diabetes and DKA. These trends are consistent with national and regional patterns that highlight the significant role of social media as an information source [24,25].

Although residency, gender, and nationality did not significantly affect knowledge levels, age emerged as a crucial factor, with younger participants (ages 18–30) showing notably higher awareness than older individuals—a trend consistent with national and regional studies [10,18,26,27]. Marital status also appeared to influence knowledge levels, as single participants exhibited a more significant percentage of good knowledge than married individuals (*p* = 0.000). Interestingly, non-working individuals displayed better overall knowledge of DKA (*p* = 0.002), which contrasts with international studies suggesting a positive correlation between employability and health literacy [28]. This could be explained by the specific socioeconomic structure of the current society, which features significant unemployment rates (43% of citizens) alongside a high level of educational attainment (about 67% with university degrees) consistent with previously published data [29].

Despite moderate awareness levels regarding DKA among participants, a knowledge gap emerged concerning the duration of diabetes. Participants without a previous history of DKA demonstrated a significantly higher percentage of poor knowledge than those who had experienced DKA (*p* = 0.020). Notably, the duration of diabetes itself did not show a significant association with levels of DKA knowledge across various duration categories, differing from findings in Switzerland, which reported a ‘significant but low positive correlation between diabetes duration and knowledge of DKA (Spearman r = 0.187; 95% CI 0.0714 to 0.298; *p* = 0.001)’ [11].

These insights into DKA awareness among patients in the Northern Borders region underscore the critical need for targeted education and intervention programs, as supported by the findings of Sathish and colleagues [30]. Efforts should focus on enhancing knowledge about DKA’s signs, symptoms, and effective management strategies, particularly among populations with lower awareness levels and those at risk due to demographics or pre-existing conditions. Consequently, fostering greater awareness could significantly improve health outcomes and reduce the incidence of DKA-related complications in this population.

## 5. Study Limitations

This study has some limitations that should be considered. First, the cross-sectional design limits our ability to establish causal relationships between demographic factors and DKA awareness. Moreover, because the data were collected through self-reported measures, there is a potential for response bias. Additionally, the non-probability convenience sampling approach used for participant selection may also restrict the generalizability of our findings, as it might not fully represent all demographics in the Northern Borders region. Lastly, external factors, such as varying access to healthcare information and resources, may further influence participants’ awareness levels and were not accounted for in this study.

## 6. Conclusions

This study highlights a critical need for public health educational interventions aimed at enhancing community knowledge regarding the management and treatment of diabetic ketoacidosis, as we identified a significant knowledge gap in this area. Furthermore, healthcare providers must play an active role in counseling and educating patients about the most common complications associated with diabetes. Relying on social media and unverified online sources for health information can adversely affect overall public health; therefore, it is essential to provide accurate, evidence-based resources to empower patients and improve their understanding of DKA and its treatment.

## Figures and Tables

**Table 1:** Baseline characteristics of the study participants.

Study Participants’ Characteristics (*n* = 339)		Frequency (*n*)	Percent (%)
Residency	Arar	267	78.8
	Outside Arar	72	21.2
Age (years)	18–30	143	42.2
	31–40	55	16.2
	41–50	70	20.6
	51–60	50	14.7
	>60	21	6.2
Sex	Male	183	54.0
	Female	156	46.0
Nationality	Saudi	320	94.4
	Non-Saudi	19	5.6
Marital status	Married	187	55.2
	Single	142	41.9
	Divorced	5	1.5
	Widowed	5	1.5
Education	Uneducated	4	1.2
	Primary	2	0.6
	Intermediate	5	1.5
	Secondary	71	20.9
	University	234	69.0
	Postgraduate	23	6.8
Occupation	I do not work	146	43.1
	Governmental institute employee	134	39.5
	Private institute employee	21	6.2
	Free business work	3	0.9
	Retired	35	10.3

Data are presented as frequencies (*n* = numbers) and percentages (%).

**Table 2:** Knowledge about diabetic ketoacidosis (DKA).

Questions	Frequency (*n*)	Percent (%)
Have you heard of diabetic ketoacidosis (DKA)? (*n* = 339)		
No	107	31.6
Yes	232	68.4
How would you describe DKA? (*n* = 232)		
An emergency complication of diabetes that requires urgent treatment.	177	76.3
One of the chronic complications of diabetes does not require a visit to a doctor; it can be treated at home.	23	9.9
It is one symptom of diabetes that I should accommodate.	32	13.8

Data are represented as frequencies and percentages (%).

**Table 3:** Knowledge about causes and contributions to DKA.

Questions	Frequency (*n*)	Percent (%)
Do you know what causes DKA? (*n* = 339)		
No	120	35.4
Yes	219	64.6
The factors you think can contribute to DKA (*n* = 219)		
Lack of insulin in the blood results from forgetting to take insulin injections.	56	25.6
High blood sugar due to irregular eating and exercise	29	13.2
All of the mentioned above	134	61.2

Data are represented as frequencies and percentages (%).

**Table 4:** Knowledge about symptoms of DKA.

Questions	No *n* (%)	Yes *n* (%)
Do you know the symptoms of DKA? (*n* = 339)	132 (38.9)	207 (61.1)
The symptoms of DKA you know (*n* = 207)		
Feeling tired/fatigue	27 (13.0)	180 (87.0)
Nausea	79 (38.2)	128 (61.8)
Unconsciousness	97 (46.9)	110 (53.1)
Stomachache	115 (55.6)	92 (44.4)
Difficulty breathing or rapid breathing	102 (30.1)	105 (31.0)
Distinctive breath smell	110 (53.1)	97 (46.9)
Blurred vision	82 (39.6)	125 (60.4)

Data are represented as frequencies and percentages (%).

**Table 5:** Knowledge about DKA treatment.

Questions	No *n* (%)	Yes *n* (%)
Do you know how DKA is treated? (*n* = 339)	127 (37.5)	212 (62.5)
Concerning treatment of DKA that you know (*n* = 212)		
Give the patient oral sugar and wait until they get better	140 (66.0)	72 (34.0)
Call an ambulance and take him to the hospital immediately	61 (28.8)	151 (71.2)
Others	179 (84.4)	33 (15.6)

Data are represented as frequencies and percentages (%).

**Table 6:** Source of information about diabetes and DKA.

Source of Information About Diabetes and DKA (*n* = 339)		Frequency (*n*)	Percent (%)
Where have you heard most of your information about diabetes and DKA?	Healthcare professional	84	24.8
	Family/friends	95	28.0
	Social media	102	30.1
	Internet	58	17.1
Do you feel you have enough information about diabetes and DKA?	No	213	62.8
	Yes	126	37.2

Data are represented as frequencies and percentages (%).

**Table 7:** Duration of reported diabetes diagnosis among study participants.

Duration of Diabetes (*n* = 339)	Frequency (*n*)	Percent (%)	Mean ± SD
≤5 years	107	31.6	11.4 ± 7.8
6–10 years	62	18.3	
11–15 years	88	26.0	
>15 years	82	24.1	

Data are represented as frequencies/percentages (%) and mean ± standard deviation (SD).

**Table 8:** Association of demographic data with overall knowledge level of DKA.

Demographic Data		Overall Knowledge Level				*p*-Value
		Poor		Good		
		*n*	%	*n*	%	
Residency	Arar	175	77.4%	92	82.1%	0.317
	Outside Arar	51	22.6%	20	17.9%	
Age (years)	18–30	76	33.6%	67	59.3%	0.000 *
	31–40	38	16.8%	17	15.0%	
	41–50	54	23.9%	16	14.2%	
	51–60	40	17.7%	10	8.8%	
	>60	18	8.0%	3	2.7%	
Sex	Male	127	56.2%	56	49.6%	0.248
	Female	99	43.8%	57	50.4%	
Nationality	Saudi	214	94.7%	106	93.8%	0.738
	Non-Saudi	12	5.3%	7	6.2%	
Marital status	Married	140	61.9%	47	41.6%	0.000 *
	Single	77	34.1%	65	57.5%	
	Divorced	5	2.2%	0	0.0%	
	Widowed	4	1.8%	1	0.9%	
Education	Uneducated	4	1.8%	0	0.0%	0.486
	Primary	2	0.9%	0	0.0%	
	Intermediate	3	1.3%	2	1.8%	
	Secondary	51	22.6%	20	17.7%	
	University	151	66.8%	83	73.5%	
	Postgraduate	15	6.6%	8	7.1%	
Occupation	I do not work	85	37.6%	61	54.0%	0.002 *
	Governmental institute employee	89	39.4%	45	39.8%	
	Private institute employee	18	8.0%	3	2.7%	
	Free business work	3	1.3%	0	0.0%	
	Retired	31	13.7%	4	3.5%	

Data are represented as numbers (*n*) and percentages (%). Chi-square and/or Fisher exact tests were applied. * The significance level was set at a *p*-value of less than 0.05.

**Table 9:** Association of duration of diabetes with overall knowledge level of DKA.

Variables		Overall Knowledge Level				*p*-Value
		Poor		Good		
		*n*	%	*n*	%	
Has a previous history of DKA	No	166	70.6%	69	29.4%	0.020 *
	Yes	60	57.7%	44	42.3%	
Duration of diabetes	≤5 years	16	48.5%	17	51.5%	0.068
	6–10 years	9	47.4%	10	52.6%	
	11–15 years	15	55.6%	12	44.4%	
	>15 years	20	80.0%	5	20.0%	

Data are represented as numbers (*n*) and percentages (%). Chi-square and/or Fisher exact tests were applied. * The significance level was set at a *p*-value of less than 0.05.

## Data Availability

The original contributions presented in this study are included in the article. Further inquiries can be directed to the corresponding authors.

## References

[1] Tangvarasittichai S. (2015). Oxidative stress, insulin resistance, dyslipidemia and type 2 diabetes mellitus. World J. Diabetes.

[2] Mohajan D., Mohajan H.K. (2023). Diabetic Ketoacidosis (DKA): A Severe Diabetes Mellitus Disorder. Stud. Soc. Sci. Humanit..

[3] Dhatariya K.K., Glaser N.S., Codner E., Umpierrez G.E. (2020). Diabetic ketoacidosis. Nat. Rev. Dis. Primers.

[4] Lizzo J.M., Goyal A., Gupta V. (2023). Adult diabetic ketoacidosis. StatPearls.

[5] Gosmanov A.R., Gosmanova E.O., Kitabchi A.E., Feingold K.R., Anawalt B., Blackman M.R. (2000). Hyperglycemic crises: Diabetic ketoacidosis and hyperglycemic hyperosmolar state. Endotext [Internet].

[6] Ahuja W., Kumar N., Kumar S., Rizwan A. (2019). Precipitating Risk Factors, Clinical Presentation, and Outcome of Diabetic Ketoacidosis in Patients with Type 1 Diabetes. Cureus.

[7] Duca L.M., Wang B., Rewers M., Rewers A. (2017). Diabetic Ketoacidosis at Diagnosis of Type 1 Diabetes Predicts Poor Long-term Glycemic Control. Diabetes Care.

[8] Lindholm Olinder A., DeAbreu M., Greene S., Haugstvedt A., Lange K., Majaliwa E.S., Pais V., Pelicand J., Town M., Mahmud F.H. (2022). ISPAD Clinical Practice Consensus Guidelines 2022: Diabetes education in children and adolescents. Pediatr. Diabetes.

[9] Ullah F., Afridi A.K., Rahim F., Ashfaq M., Khan S., Shabbier G., Rahman S.U. (2015). Knowledge of Diabetic Complications in Patients With Diabetes Mellitus. J. Ayub Med. Coll. Abbottabad.

[10] Thakare P.S., Ankar R. (2021). To assess the knowledge regarding signs and symptoms of diabetic ketoacidosis and its prevention among diabetes patients in Wardha District, Maharashtra, India. J. Evol. Med. Dent. Sci..

[11] Hepprich M., Roser P., Stiebitz S., Felix B., Schultes B., Schmitz D., Rutishauser J., Schubert S., Aberle J., Rudofsky G. (2023). Awareness and knowledge of diabetic ketoacidosis in people with type 1 diabetes: A cross-sectional, multicenter survey. BMJ Open Diabetes Res. Care.

[12] Elhassan A.B.E., Saad M.M.E., Salman M.S.T., Ibrahim A., Ali A., Alla A., Saad F.M. (2022). Diabetic ketoacidosis: Knowledge and practice among patients with diabetes attending three specialized diabetes clinics in Khartoum, Sudan. Pan Afr. Med. J..

[13] Alışkan H., Kılıç M. (2022). The relationship between initial lactate levels and outcomes in patients diagnosed with diabetic ketoacidosis in the emergency department: Initial lactate levels and outcomes in patients with diabetic ketoacidosis. J. Surg. Med..

[14] Farran B.A., Bin Elaiwah R.I., Aldarsouny A.T., Alshamrani A.M., Almaslamani A.M., Alsubie B.F., Zainab M.M., Alkulaib M.O., Khalifah A. (2020). Level of awareness of diabetic ketoacidosis among diabetes mellitus patients in Riyadh. J. Fam. Med. Prim. Care.

[15] Hassan A., Alhuthaili A., Mudawi M., Elamin M., Atia T.H., Alshinqiti M., Alfawaz K., Alamri M., Sufyani A., Alasmari R.M. (2024). The Knowledge, Attitudes, and Practices Regarding Diabetic Ketoacidosis Among Diabetic Patients in the Northern and Western Regions of Saudi Arabia. Cureus.

[16] Al Kaabba A.F., Alzuair B.S., AlHarbi Y.F., Alshehri J.A., Allowaihiq L.H., Alrashid M.H., Alkhatabi R.A. (2021). Knowledge and awareness of caregivers about diabetic ketoacidosis among type-1 diabetic children and their action and response in Riyadh City. Open J. Endocr. Metab. Dis..

[17] Eltom E.H., Alanazi A.L., Alenezi J.F., Alruwaili G.M., Alanazi A.M., Hamayun R. (2022). Self-medication with antibiotics and awareness of antibiotic resistance among population in Arar city, Saudi Arabia. J. Infect. Dev. Ctries..

[18] Alsaedi A.A., Alsaedi M.A., Eterji A.S., Alshenqity A.A., Alshenqity M.A., Alsaedi R.A., Alsaedi R.A., Alsaedi Z.A., Alsulami B.K., Shatla M.M. (2024). The Assessment of Diabetic Ketoacidosis Awareness Among Diabetic Patients and Their Caregivers in Makkah, Saudi Arabia: A Cross-Sectional Study. Cureus.

[19] Nazar C.M., Bojerenu M.M., Safdar M., Marwat J. (2016). Effectiveness of diabetes education and awareness of diabetes mellitus in combating diabetes in the United Kigdom; a literature review. J. Nephropharmacol..

[20] Świątoniowska N., Sarzyńska K., Szymańska-Chabowska A., Jankowska-Polańska B. (2019). The role of education in type 2 diabetes treatment. Diabetes Res. Clin. Pract..

[21] Ernawati U., Wihastuti T.A., Utami Y.W. (2021). Effectiveness of diabetes self-management education (DSME) in type 2 diabetes mellitus (T2DM) patients: Systematic literature review. J. Public Health Res..

[22] Raveendran A.V., Chacko E.C., Pappachan J.M. (2018). Non-pharmacological Treatment Options in the Management of Diabetes Mellitus. Eur. Endocrinol..

[23] Mencher S.R., Frank G., Fishbein J. (2019). Diabetic Ketoacidosis at Onset of Type 1 Diabetes: Rates and Risk Factors Today to 15 Years Ago. Glob. Pediatr. Health.

[24] Abu-Farha R., Mukattash T., Itani R., Karout S., Khojah H.M.J., Abed Al-Mahmood A., Alzoubi K.H. (2021). Willingness of Middle Eastern public to receive COVID-19 vaccines. Saudi Pharm. J. SPJ Off. Publ. Saudi Pharm. Soc..

[25] Alduraywish S.A., Altamimi L.A., Aldhuwayhi R.A., AlZamil L.R., Alzeghayer L.Y., Alsaleh F.S., Aldakheel F.M., Tharkar S. (2020). Sources of Health Information and Their Impacts on Medical Knowledge Perception Among the Saudi Arabian Population: Cross-Sectional Study. J. Med. Internet Res..

[26] Alruwaili A.F., AlArjan F.M., Alruwaili A.S.K., Almulhim F.A., Alenazi A.H., Aldandani R.R.A., Alanzi A.M.A., Alfuhigi F.R., Alanazi A.O.A. (2018). Awareness, Frequency and prevalence of DKA with DM type 1 children in Al-Jouf Region. Egypt. J. Hosp. Med..

[27] Hamed N.F., Alhawiti M.M.E., Albalawi E.H.A., Alzahrani L.D.G., Alhawiti R.M.E., Alatawi S.Y.S., Albalawi M.A.M., Albalawi B.M.M., Alzahrani A.A.M., Alsayed M.S.A. (2021). Awareness of parents regarding DKA symptoms in their children with type I DM. J. Pharm. Res. Int..

[28] Ehmann A.T., Ög E., Rieger M.A., Siegel A. (2021). Work-Related Health Literacy: A Scoping Review to Clarify the Concept. Int. J. Environ. Res. Public Health.

[29] Alnuaim M. (2013). The Composition of the Saudi Middle Class: A Preliminary Study.

[30] Sathish T., Thankappan K.R., Panniyammakal J., Oldenburg B. (2023). Knowledge of Diabetes among Adults at High Risk for Type 2 Diabetes in the Trivandrum District of Kerala, India. Diabetology.

